# Health-Related Quality of Life and Medical Resource Use in Patients with Osteoporosis and Depression: A Cross-Sectional Analysis from the National Health and Nutrition Examination Survey

**DOI:** 10.3390/ijerph17031124

**Published:** 2020-02-10

**Authors:** Shih-Feng Weng, Hui-Ru Hsu, Yao-Lin Weng, Kai-Jen Tien, Hao-Yun Kao

**Affiliations:** 1 Department of Healthcare Administration and Medical Informatics, College of Health Sciences, Kaohsiung Medical University, Kaohsiung 80708, Taiwan; sfweng@kmu.edu.tw; 2 Department of Medical Research, Kaohsiung Medical University Hospital, Kaohsiung 80708, Taiwan; 3 Center for Medical informatics and Statistics, Office of R&D, Kaohsiung Medical University, Kaohsiung 80708, Taiwan; 4 Division of Financial Management, Kaohsiung Medical University Hospital, Kaohsiung 80708, Taiwan; e7162721ab@yahoo.com.tw; 5 Department of Food and Nutrition, Providence University, Taichung 43301, Taiwan; ylweng@pu.edu.tw; 6 Division of Endocrinology and Metabolism, Department of Internal Medicine, Chi Mei Medical Center, Tainan 71004, Taiwan

**Keywords:** osteoporosis, depression, medical resource use, health-related quality of life, NHANES

## Abstract

*Background*: Patients with either osteoporosis or depression are prone to develop other diseases and require more medical resources than do the general population. However, there are no studies on health-related quality of life (HRQoL) and medical resource use by osteoporosis patients with comorbid depression. We conducted this study for clarifying it. *Methods*: This cross-sectional study from 2005 to 2010 (6 years) analyzed 9776 National Health and Nutrition Examination Survey (NHANES) patients > 40 years old. Each patient was assigned to one of four groups: osteoporosis-positive^(+)^ and depression-positive^(+)^ (O^+^/D^+^); O^+^/D^−^; O^−^/D^+^; O^−^/D^−^. We used multivariate linear and logistic regression model to analyze the HRQoL and medical resource use between groups. *Results*: The O^+^/D^+^ group reported more unhealthy days of physical health, more unhealthy days of mental health, and more inactive days during a specified 30 days. The adjusted odds ratios (AORs) of O^+^/D^+^ patients who had poor general health (7.40, 95% CI = 4.80–11.40), who needed healthcare (3.25, 95% CI = 2.12–5.00), and who had been hospitalized overnight (2.71, 95% CI = 1.89–3.90) were significantly highest. *Conclusions*: Low HRQoL was significantly more prevalent in D^+^/O^+^ patients. We found that depression severity more significantly affected HRQoL than did osteoporosis. However, both diseases significantly increased the risk of high medical resource use.

## 1. Introduction

Osteoporosis is a common skeletal disorder that leads to a systemic reduction of bone mass, strength, and skeletal microarchitecture [1], and it significantly increases the risk of fractures. The subsequent loss of mobility often causes a major reduction in health-related quality of life (HRQoL). The World Health Organization (WHO, Geneva, Switzerland) reported that osteoporosis is a major cause of serious health problems and increases mortality in elderly patients. In 2010, about 22 million women and 5.5 million men in the European Union had osteoporosis [2]. About 8 million women and one to two million men had osteoporosis in the United States (USA) in 2012 [3], and the prevalence is expected to increase in the near future. In the developing world, 2% to 8% of men and 9% to 38% of women had osteoporosis [4].

An association between major depressive disorder (MDD) and osteoporosis has been reported: patients with MDD apparently have significantly lower bone mineral density (BMD) [5]. Possible factors of reduced BMD in depression patients are the hypothalamic-pituitary-adrenal (HPA) axis: cortisol, leptin, and immune factor levels; cytokine, vitamin D, and parathyroid hormone levels; gender, lifestyle factors, the effect of antidepressants on BMD, osteoporotic fractures, and other comorbid psychiatric conditions [6]. Depression may increase serum cortisol level via activating the hypothalamic corticotropin-releasing hormone neuron. Hypercortisolemia is considered to be a destructive factor for bone health [6]. Depressed patients may have elevated leptin levels, which cause bone loss and inhibits bone formation via activation of sympathetic systems [7,8]. Depression is associated with immune dysregulation and increased oxidative stress, which stimulates HPA axis and results in hypercortisolemia [9,10]. Besides, depression is associated with lower vitamin D and increased parathyroid hormone levels, which may impact bone remodeling [11]. Antidepressants may interfere with sex hormone production such as androgen, which may decrease bone mass and increase risk of orthostatic hypotension, falls and bone fractures [12,13,14]. The incidence rate of depression or anxiety in women with osteoporosis was 46.8 per 1000 person years in the USA [15]. One study utilized data from US households civilian population from 2007 to 2009, claimed that comorbid depression and anxiety have 2.47 times risk of osteoporosis [16]. Approximately 45% of women with depression had osteoporosis, the duration of their depression was strongly negatively correlated with their BMD, and the depression was probably associated with reduced physical activity [17]. Moreover, depressed patients have higher predictive risk of hip fracture and high depressive symptomatology remained predictive of higher risk of hip fracture than those with low depressive symptomatology [18]. Furthermore, depression appears to increase comorbid medical conditions, disabilities, and healthcare resource use, and to reduce HRQoL [19].

Osteoporosis^+^ (O^+^) patients with depression and a low HRQoL have a high risk of poor long-term surgical outcomes [20,21,22]. Most studies of the medical resources used focus on osteoporotic fractures; femoral fractures use the most resources because patients require surgery and hospitalization. Because the population is aging and the prevalence of fractures is increasing, we must develop osteoporosis management strategies that reduce the healthcare burden [23]. Osteoporosis^+^ patients have higher levels of comorbid anxiety, depression, chronic somatic non-musculoskeletal diseases, and pain than do O^−^ (osteoporosis-negative) patients. The problems associated with activities of daily living (ADLs), intense pain, anxiety, and depression might increase a patient’s need for medical resources (number of healthcare visits/year, hospitalized overnight in the past year) [24].

Patients with either osteoporosis or depression are prone to develop other diseases and require more medical resources than do the general population. However, there are no studies on HRQoL and medical resource use to explored how (1) depression only and (2) osteoporosis only and (3) depression and osteoporosis combined affected the perception of quality of life and the use of medical resources; therefore, in this study we explored this topic.

## 2. Methods

The National Health and Nutrition Examination Survey (NHANES) is a cross-sectional survey conducted by the Centers for Disease Control and Prevention’s (CDC, Atlanta, GA, USA) National Center for Health Statistics (NCHS, Hyattsville, MD, USA). NHANES collects data on the health and nutritional status of the USA’s adults and children. We used the data from 2005 to 2010 of patients > 40 years old [25,26]). Patients with missing BMD data and incomplete HRQoL information were excluded. In our study, each patient was assigned to one of four groups: O^+^/D^+^; O^−^/D^+^; O^+^/D^−^; or O^−^/D^−^.

In NHANES, depression was measured using the Patient Health Questionnaire (PHQ-9), a nine-item screening instrument that asks questions (scored from 0 [not at all] to 3 [nearly every day]; total score: 0–27) about the frequency of symptoms of depression over the previous 2 weeks. A score ≥ 10, commonly used in clinical studies to define depression, has been well validated. Whether a patient was diagnosed as O^+^ or O^−^ was based on the dual-energy X-ray absorptiometry (DXA)-determined BMD of the total femur, femoral neck, trochanter, intertrochanter, Ward’s triangle, total spine, and vertebrae L1–L4. Patients were diagnosed as O^+^ or O^−^ if one of three criteria was met: (1) femur neck BMD < 0.558 g/cm^2^, (Cunningham [27]) (2) T-score < −2.5 standard deviations (SDs), (3) patient said “yes” to the question: “Has a doctor ever told you that you had osteoporosis, sometimes called ‘thin or brittle bones’?” (T-score = [BMD − reference BMD]/reference SD; reference group: 20- to 29-year-old non-Hispanic white women).

Health-related quality of life (HRQoL) is useful because it provides information on an individual’s physical and mental health status and on its effect on their QoL [28]. The HRQoL-4 tool, which was developed by the CDC, includes four questions: (1) “How many days during the past 30 days was your physical health not good?”; (2) “How many days during the past 30 days was your mental health not good?”; (3) “How many days during the past 30 days did pain make it hard for you to do usual activities, such as self-care, work, or recreation?”; (4) “Would you say your health in general is: excellent, very good, good, fair, or poor?”.

The three measurements of hospital use and access to healthcare in this study were obtained from answers to the Hospital Utilization & Access to Care questionnaire (HUQ_G) in NHANES: (1) What kind of place do you routinely go to for healthcare? This had three possible answers: a clinic or health center, a doctor’s office or health maintenance organization (HMO), or others; (2) the number of healthcare visits/year was categorized into two categories: 0–3 visits and > 4 visits; (3) hospitalized overnight last year was categorized into yes or no.

Demographic characteristics included gender, age, race (non-Hispanic white, non-Hispanic black, Mexican American or other Hispanic, other race [including multiracial]), education (<high school, high school or equivalent, college graduate or above), marital status (married, never married, widowed, living with partner, or separated or divorced), and the ratio of family income to poverty (RIP). Health condition variables include body mass index (BMI: < 18.5, 18.5–25.0, ≥ 25.0), cigarette smoking, alcohol drinking, sleep disorders, hypertension, and diabetes.

The NHANES is a publicly available database and approved by the National Center for Health Statistics institutional review board. All participants provide written informed consent. This study was approved by the Institutional Review Board of Kaohsiung Medical University Hospital (KMUHIRB-EXEMPT(I)-20170030).

### Statistical Analysis

Descriptive statistics were used to compare the characteristics of disease groups. The χ^2^ test was used for categorical variables, and the analysis of variance (ANOVA) was used for continuous variables. Multivariate linear regressions were used to analyze physically unhealthy days, mentally unhealthy days, and limited activity days. Multivariate logistic regressions were used to analyze hospital use and health conditions between groups after gender, age, race, education, marital status, the RIP, BMI, cigarette smoking, alcohol drinking, sleep disorders, hypertension, and diabetes had been adjusted for. SAS 9.4 (SAS Institute Inc., Cary, NC, USA) was used to analyze the data. Significance was set at *p* < 0.05.

## 3. Results

**Figure 1:**
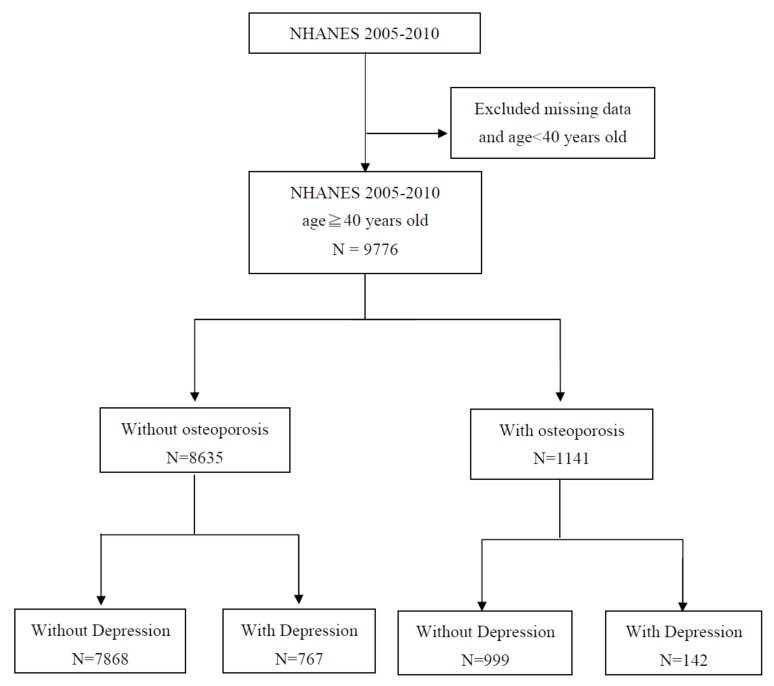
Flow chart of this study.

Of the 9776 patients evaluated in this study, 7868 (80.48%) were O^−^/D^−^, 999 (10.22%) were O^+^/D^−^, 767 (7.85%) were O^−^/D^+^, and 142 (1.45%) were O^+^/D^+^ (Table 1). The flow chart of this study was performed, as shown in Figure 1. Osteoporosis^+^/depression^+^ patients were more likely to be female (84.51%), to be widowed, separated, or divorced (57.05%), to be less educated (50.70%), to be Hispanic (Mexican American or other) (32.39%), and to have a lower RIP than did other patients. They were more likely to smoke cigarettes (64.79%), to drink less alcohol (50.70%), and to have more hypertension (66.90%), sleep disorders (32.39%), and diabetes (32.39%) than did other patients.

Osteoporosis^(+)^/depression^(+)^ patients had significantly (*p* < 0.001) more totally unhealthy days (23.33 ± 9.94), mentally unhealthy days (17.82 ± 16.78), physically unhealthy days (16.78 ± 15.42), and days of limited activity (12.85 ± 14.01) in the previous 30 days than did other patients (Table 2). More of them reported that their health was fair (40.14%) or poor (36.42%) and that they had more than four healthcare visits per year (79.58%), and more of them (39.44%) said that they had been hospitalized overnight often in the previous year. All patients in all groups said that they often went to a doctor’s office or an HMO for healthcare in the previous year.

Table 3 indicates that D^+^ patients had significantly (*p* < 0.001) more days during which they were physically and mentally unhealthy, and significantly more days when their activity was limited than did D^−^ patients. Osteoporosis^+^/depression^+^ group patients had more days that were physically (β = 10.94), mentally unhealthy (β = 14.13), and totally unhealthy days (β = 15.13), and more days of limited activity (β = 10.25) than did O^−^/D^−^ group patients. However, only O^+^/D^−^ patients had fewer mentally unhealthy days than did O^−^/D^−^ patients.

The group risks for fair or poor health, after gender, age, race, education, marital status, the RIP, cigarette smoking, alcohol drinking, sleep disorders, hypertension, and diabetes had been adjusted for were O^+^/D^+^ (AOR: 7.40; 95% CI: 4.80–11.4; *p* < 0.001), O^−^/D^+^ (AOR: 4.79; 95% CI: 4.02–5.72; *p* < 0.001), and O^+^/D^−^ (AOR: 1.61; 95% CI: 1.35–1.91; *p* < 0.001) than was that of the O^−^/D^−^ group (Table 4).

There was no significant difference of medical resource use between groups in visiting the doctor’s office or HMOs (Table 5). The group risks for a greater number of healthcare visits (>four times in the previous year) after adjustment were O^+^/D^+^ (AOR: 3.25; 95% CI: 2.12–5.00; *p* < 0.001), O^−^/D^+^ (AOR: 2.09; 95% CI: 1.76–2.47; *p* < 0.001), and O^+^/D^−^ (AOR: 1.57; 95% CI: 1.35–1.82; *p* < 0.001) than was that of the O^−^/D^−^ group. In addition, the group risks to be hospitalized overnight in the previous year after adjustment were O^+^/D^+^ (AOR: 2.71; 95% CI: 1.89–3.90; *p* < 0.001), O^−^/D^+^ (AOR: 1.80; 95% CI: 1.48–2.18; *p* < 0.001), and O^+^/D^−^ (AOR: 1.32; 95% CI: 1.10–1.59; *p* < 0.003) than was that of the O^−^/D^−^ group (Table 5).

## 4. Discussion

This is the first study that examines the HRQoL and medical resource use of patients with osteoporosis and depression. After we reviewed the data of a nationally representative sample of the USA’s population, we found that depression severity more significantly affected HRQoL than did osteoporosis. However, both diseases significantly affect medical resource use: O^+^/D^+^ patients reported more healthcare visits overnight hospitalizations during the previous year, and worse health during the NHANES study.

In our study, although O^+^ patients had worse HRQoL than did O^−^ patients, HRQoL was significantly more negatively affected in D^+^ patients. We found that being D^+^ was associated with more physically and mentally unhealthy days and with more limited activity days than was being O^+^. This is consistent with many other studies [29,30,31,32,33]. It also indicates that O^+^ patients might not develop clinical symptoms until their bones become so fragile that a sudden strain, bump, or fall causes a bone fracture. This is why osteoporosis is often called “the silent thief” [34]. Reduced HRQoL in patients with osteoporosis is caused primarily by fractures, particularly of the spine or hip, which cause pain and impair physical, social, and mental function [35,36,37]. Therefore, many HRQoL instruments that measure the effects of osteoporosis focus on vertebral, hip, and other nontraumatic fractures on patient function and psychosocial well-being [38,39], and find poor HRQoL after fractures [22]. However, although the primary O^+^ symptom is fracture, other problems, (e.g., the RIP, education, chronic pain, and cigarette smoking) reduce physical capacity and function, and depression worsens the HRQoL of O^+^ patients [20,40].

It is well-known that having a regular doctor or some other usual source of healthcare is important to health outcomes [41,42,43]. Although other studies reported that O^+^ patients go most often to a doctor’s office or HMO more than to clinic or health center [44], we found no significant difference in the type of routine healthcare provider visited. This might be explained by the absence of a universal national healthcare provider in the USA, by the extraordinarily wide range of prices for even routine healthcare treatments, by the inability of American residents to afford healthcare, or by a combination of some or all of these reasons. There is growing interest in investigating the effects of the currently chaotic and volatile healthcare environment in the USA on healthcare and outcomes there [45,46].

Other studies [33,47,48] have hypothesized that of depression is significantly related to using medical resources. There is also evidence from other studies [24,44] which shows that O^+^ patients are more likely to have visited a general practitioner’s office in the previous four weeks, to make ≥ 4 healthcare visits/year, and to have been hospitalized overnight in the previous year. Our study demonstrated that being both O^+^ and D^+^ is positively associated with the frequency of using healthcare providers and medical resources, and with healthcare outcomes.

An Austrian Health Interview Survey [24] said that most medical resources used by O^+^ patients > 65 years old was affected by depression, which is consistent with our findings. Therefore, effectively screening and managing depression should reduce the costs and the overall use of medical resources, and it should improve healthcare outcome.

### Strengths and Limitations

The strengths of our study are that we used population-based data of O^+^ and D^+^ patients ≥ 40 years old, and that our statistical models were controlled for possible confounding sociodemographic, anthropometric, and health variables. Our study also has some limitations. First, the data were from a discontinuous eight-year NHANES study. Second, the NHANES is a cross-sectional rather than a longitudinal cohort study; thus, it is not possible to infer causality. Third, the follow-up for all patients was only a little longer than two years. Fourth, HRQoL and medical resource use were self-reported data, but patients might not have understood the questions, or patients might have been affected by a social desirability bias [49]. Fifth, the severity of depression was not stated for each patient, but different severity levels might significantly affect HRQoL and medical resource use. Sixth, the types and quality of medications used to treat osteoporosis and depression have not been evaluated, but they might affect HRQoL and medical resource use. Seventh, the depressive symptoms of more than half the USA’s NHANES participants had not been treated, and about three-quarters of them had severe depression but did not take antidepressants [50].

## 5. Conclusions

HRQoL was significantly more affected by depression than by osteoporosis. Osteoporosis^+^ patients comorbid with depression were likely to have required more medical resources in the previous year, and to have felt that their health was poorer than was that of O^+^/D^−^, O^−^/D^+^, and O^−^/D^−^ patients. Therefore, it is important to focus on preventing O^+^ patients from developing depression. This study implicates that it is necessary to consider the psychological status when the clinicians approach osteoporotic patients in clinical practice. Early detection and intervention of depression in osteoporotic patients may have the opportunity to improve HRQoL and reduce the use of medical resources.

## Figures and Tables

**Table 1:** Characteristics of four groups.

Variable	O^−^/D^−^	O^+^/D^−^	O^−^/D^+^	O^+^/D^+^	*p*
Total, n (%)	7868 (80.48)	999 (10.22)	767 (7.85)	142 (1.45)	
Year					
2005–2006	2223 (83.04)	251 (6.28)	168 (9.38)	35 (1.3)	0.003
2007–2008	2798 (79.11)	387 (8.31)	294 (10.94)	58 (1.64)	
2009–2010	2847 (79.93)	361 (8.56)	305 (10.13)	49 (1.38)	
Demographic					
Gender					
Male	4314 (54.83)	201 (20.12)	333 (43.42)	22 (15.49)	<0.001
Female	3554 (45.17)	798 (79.88)	434 (56.58)	120 (84.51)	
Age	59.15 ± 12.42	69.53 ± 10.69	55.88 ± 11.22	64.01 ± 11.21	<0.001
Race					
Non-Hispanic white	4042 (51.37)	649 (64.96)	349 (45.50)	75 (52.82)	<0.001
Non-Hispanic black	1653 (21.01)	114 (11.41)	168 (21.90)	16 (11.27)	
Hispanic (Mexican American or other)	1908 (24.25)	200 (20.02)	218 (28.42)	46 (32.39)	
Other (including multiracial)	265 (3.37)	36 (3.60)	32 (4.17)	5 (3.52)	
Education					
<High school	2293 (29.14)	321 (32.13)	328 (42.76)	72 (50.70)	<0.001
High school or equivalent	1858 (23.61)	267 (26.73)	174 (22.69)	34 (23.94)	
College graduate or above	3717 (47.24)	411 (41.14)	265 (34.55)	36 (25.35)	
Marital Status					
Married	4844 (61.57)	496 (49.65)	355 (43.68)	50 (35.21)	<0.001
Never married	532 (6.76)	44 (4.40)	80 (10.43)	11 (7.75)	
Widowed	843 (10.71)	301 (30.13)	90 (11.73)	37 (26.06)	
Living with partner, separated, or divorced	1649 (20.96)	158 (15.82)	262 (34.16)	44 (30.99)	
Family poverty index ratio (RIP)	2.79 ± 1.56	2.47 ± 1.44	1.89 ± 1.37	1.76 ± 1.22	<0.001
General health condition					
Body mass index, kg/m^2^					
<18.5	88 (1.12)	28 (2.80)	13 (1.69)	2 (1.41)	<0.001
18.5–25.0	1705 (21.67)	392 (39.24)	150 (19.56)	41 (28.87)	
≥25.0	6075 (77.21)	579 (57.96)	604 (78.75)	99 (69.72)	
Cigarette smoking	3973 (50.50)	473 (47.35)	469 (61.15)	92 (64.79)	<0.001
Alcohol drinking	5550 (70.54)	560 (56.06)	525 (68.45)	72 (50.70)	<0.001
Sleep disorders	604 (7.68)	82 (8.21)	179 (23.34)	46 (32.39)	<0.001
Hypertension	3560 (45.25)	540 (54.05)	413 (53.85)	95 (66.90)	<0.001
Diabetes	1232 (15.66)	168 (16.82)	172 (22.43)	46 (32.39)	<0.001

Data are expressed as n (%) or as mean ± standard deviation (SD).

**Table 2:** The distributions of HRQoL and hospital use between disease groups.

Variable	O^−^/D^−^	O^+^/D^−^	O^−^/D^+^	O^+^/D^+^	*p*
Physically unhealthy days ^†^	3.90 ± 8.87	5.67 ± 11.25	13.93 ± 13.31	16.78 ± 15.42	<0.001
Mentally unhealthy days ^‡^	2.60 ± 7.21	3.20 ± 8.15	17.61 ± 14.15	17.82 ± 16.78	<0.001
Totally unhealthy days ^#^	5.85 ± 9.51	7.62 ± 10.73	22.73 ± 10.59	23.33 ± 9.94	<0.001
Limited activity days ^§^	1.63 ± 6.64	2.65 ± 8.52	10.79 ± 12.94	12.85 ± 14.01	<0.001
Health status					
Excellent	729 (9.27)	66 (6.61)	11 (1.43)	0 (0.00)	<0.001
Very good	2183 (27.75)	236 (23.62)	50 (6.52)	4 (2.82)	
Good	3156 (40.11)	383 (38.34)	212 (27.64)	29 (20.42)	
Fair	1587 (20.17)	256 (25.63)	325 (42.37)	57 (40.14)	
Poor	213 (2.71)	58 (5.81)	169 (22.03)	52 (36.42)	
Type of routine healthcare place visited					
Clinic, health center, or others	2689 (34.18)	248 (24.82)	328 (42.76)	55 (38.13)	<0.001
Doctor’s office or HMO	5179 (65.82)	751 (75.18)	439 (57.24)	87 (61.27)	
Number of healthcare visits/year					
0–3 visits	4521 (57.46)	379 (37.94)	299 (38.98)	29 (20.42)	<0.001
≥4 visits	3347 (42.54)	620 (62.06)	468 (61.02)	113 (79.58)	
Times hospitalized overnight previous year					
Yes	993 (12.62)	200 (20.02)	182 (23.73)	56 (39.44)	<0.001
No	6875 (87.38)	799 (79.98)	585 (76.27)	86 (60.56)	

Data are expressed as n (%) or as mean ± standard deviation (SD). ^†^ Number of days during the preceding 30 days when physical health, including physical illness or injury, was not good. ^‡^ Number of days during the preceding 30 days when mental health, including stress, depression, or emotional problems, was not good. ^#^ Number of days during the preceding 30 days when physical health or mental health was not good. For someone who reports 30 physically unhealthy days and 30 mentally unhealthy days is assigned the maximum of 30 unhealthy days. ^§^ Number of days during the preceding 30 days when usual activities, including self-care, work, or recreation, were limited.

**Table 3:** Linear regression analysis of HRQoL.

	Univaritate		Multivariate *	
	Beta	*p*	Beta	*p*
Physically unhealthy days				
O^+^/D^+^	11.90	<0.001	10.94	<0.001
O^−^/D^+^	9.63	<0.001	9.02	<0.001
O^+^/D^−^	0.69	0.041	1.17	<0.001
O^−^/D^−^	reference		reference	
Mentally unhealthy days				
O^+^/D^+^	13.96	<0.001	14.13	<0.001
O^−^/D^+^	14.70	<0.001	14.11	<0.001
O^+^/D^−^	−0.96	0.002	0.48	0.105
O^−^/D^−^	reference		reference	
Totally unhealthy days			
O^+^/D^+^	15.95	<0.001	15.13	<0.001
O^−^/D^+^	16.41	<0.001	15.28	<0.001
O^+^/D^−^	0.01	0.976	1.38	<0.001
O^−^/D^−^	reference		reference	
Activity limitation days				
O^+^/D^+^	10.38	<0.001	10.25	<0.001
O^−^/D^+^	8.88	<0.001	8.58	<0.001
O^+^/D^−^	0.04	0.885	0.83	0.002
O^−^/D^−^	reference		reference	

Data are expressed as n (%) or as mean ± SD. * Adjusted for gender, age, race, education, marital status, the RIP, BMI, cigarette smoking, alcohol drinking, sleep disorders, hypertension, and diabetes.

**Table 4:** Logistic regression to compare the risk for fair and poor HRQoL between disease groups.

	OR (95% CI)	*p*	AOR (95% CI) *	*p*
O^+^/D^+^	8.90 (6.01–13.17)	<0.001	7.40 (4.80–11.40)	<0.001
O^−^/D^+^	5.52 (4.73–6.45)	<0.001	4.79 (4.02–5.72)	<0.001
O^+^/D^−^	1.22 (1.06–1.40)	0.007	1.61 (1.35–1.91)	<0.001
O^−^/D^−^	1.00		1.00	

OR, odds ratio; AOR, adjusted odds ratio; CI, confidence interval. * Adjusted for gender, age, race, education, marital status, the RIP, BMI, cigarette smoking, alcohol drinking, sleep disorders, hypertension, and diabetes.

**Table 5:** Comparison of the risk for medical resource utilization among disease groups by unconditional logistic regression.

	OR (95% CI)	*p*-Value	AOR (95% CI) *	*p*-Value
Doctor’s office or HMO				
O^+^/D^+^	0.81 (0.58, 1.14)	0.227	0.88 (0.61, 1.26)	0.473
O^−^/D^+^	0.67 (0.57, 0.77)	<0.001	0.96 (0.81, 1.13)	0.622
O^+^/D^−^	1.63 (1.40, 1.89)	<0.001	1.15 (0.97, 1.36)	0.107
O^−^/D^−^	1.00		1.00	
Healthcare visits ≥4/year				
O^+^/D^+^	4.57 (3.03, 6.88)	<0.001	3.25 (2.12, 5.00)	<0.001
O^−^/D^+^	1.89 (1.63, 2.20)	<0.001	2.09 (1.76, 2.47)	<0.001
O^+^/D^−^	2.02 (1.77, 2.31)	<0.001	1.57 (1.35, 1.82)	<0.001
O^−^/D^−^	1.00		1.00	
Hospitalized overnight last year				
O^+^/D^+^	3.91 (2.78, 5.50)	<0.001	2.71 (1.89, 3.90)	<0.001
O^−^/D^+^	1.93 (1.62, 2.31)	<0.001	1.80 (1.48, 2.18)	<0.001
O^+^/D^−^	1.53 (1.30, 1.81)	<0.001	1.32 (1.10, 1.59)	0.003
O^−^/D^−^	1.00		1.00	

OR, odds ratio; AOR, adjusted odds ratio; CI, confidence interval. * Adjusted for gender, age, race, education, marital status, RIP, BMI, cigarette smoking, alcohol drinking, sleep disorders, hypertension, and diabetes.

## Abbreviations

NHANES	National Health and Nutrition Examination Survey
HRQoL	health-related quality of life
O^+^	osteoporosis-positive^(+)^
O^−^	osteoporosis-negative^(−)^
D^+^	epression-positive^(+)^
D^−^	epression-negative^(−)^
OR	odds ratio
AOR	adjusted odds ratio
CI	confidence interval
MDD	major depressive disorder
BMD	bone mineral density
ADLs	activities of daily living (ADLs)
PHQ-9	Patient Health Questionnaire
CDC	Centers for Disease Control and Prevention
NCHS	National Center for Health Statistics
RIP	ratio of family income to poverty
BMI	body mass index
